# Chemical tools for decoding redox signaling at the host–microbe interface

**DOI:** 10.1371/journal.ppat.1009070

**Published:** 2020-12-17

**Authors:** Elizabeth M. Gordon, Stavroula K. Hatzios

**Affiliations:** 1 Department of Molecular, Cellular, and Developmental Biology, Yale University, New Haven, Connecticut, United States of America; 2 Microbial Sciences Institute, Yale University, West Haven, Connecticut, United States of America; 3 Department of Chemistry, Yale University, New Haven, Connecticut, United States of America; University of Massachusetts, Worcester, UNITED STATES

## Introduction

Host cells deploy a variety of chemically reactive small molecules, such as reactive oxygen species (ROS) and reactive nitrogen species (RNS), to defend themselves against invading pathogens. These molecules are primarily known for their bactericidal activity; however, oxidants like hydrogen peroxide and nitric oxide can also function as signaling molecules that posttranslationally modify redox-sensitive amino acids under non-pathological conditions [1,2]. Mounting evidence suggests that these oxidants represent an important line of communication between bacterial and host cells. Although it has historically been difficult to detect such redox-signaling events, recently developed chemical proteomic techniques have made it possible to resolve precise oxidation sites within complex proteomes. Here, we focus specifically on the role of ROS as chemical messengers at the host–microbe interface and highlight new technologies for decoding ROS-mediated signaling. Given that ROS generation is an important factor in many infection-associated pathologies [3], a deeper understanding of how these molecules influence cell signaling during infection could have important implications for human health and disease.

## How are ROS generated at the host–microbe interface?

Redox signaling—the regulation of cell signaling by oxidative posttranslational modifications (OxiPTMs)—modulates diverse cellular processes including cell division, metabolism, and antioxidant defense [2]. In mammalian systems, redox signaling is typically activated by ROS produced in response to external stimuli such as growth factors, cytokines, and microbial contact with epithelial barriers [1,2]. Hydrogen peroxide and superoxide are considered the primary agents of ROS-mediated signaling. NADPH oxidases (NOX) synthesize hydrogen peroxide and superoxide in response to extracellular signals [2]. NOX2, an isoform expressed by phagocytic cells, catalyzes the oxidative burst that is used to eliminate invading pathogens. Other NOX isoforms are expressed by different cell types and produce non-pathological levels of ROS that also contribute to mucosal defense [2].

As in mammalian cells, numerous biological processes contribute to ROS production in bacteria. The most common sources of bacterial ROS are cellular respiration and the autoxidation of flavoproteins [1,4]. A recently discovered class of NOX-like enzymes in bacteria may also catalyze ROS formation [5]. In addition, certain commensal and pathogenic bacteria excrete millimolar levels of hydrogen peroxide generated by specialized oxidases [6,7]. Pathogens can also secrete redox-active metabolites that cause damage to epithelial tissues [8], suggesting that microbe-derived ROS may also regulate redox signaling in the host.

## How do ROS modulate cell signaling at the posttranslational level?

**Fig 1:**
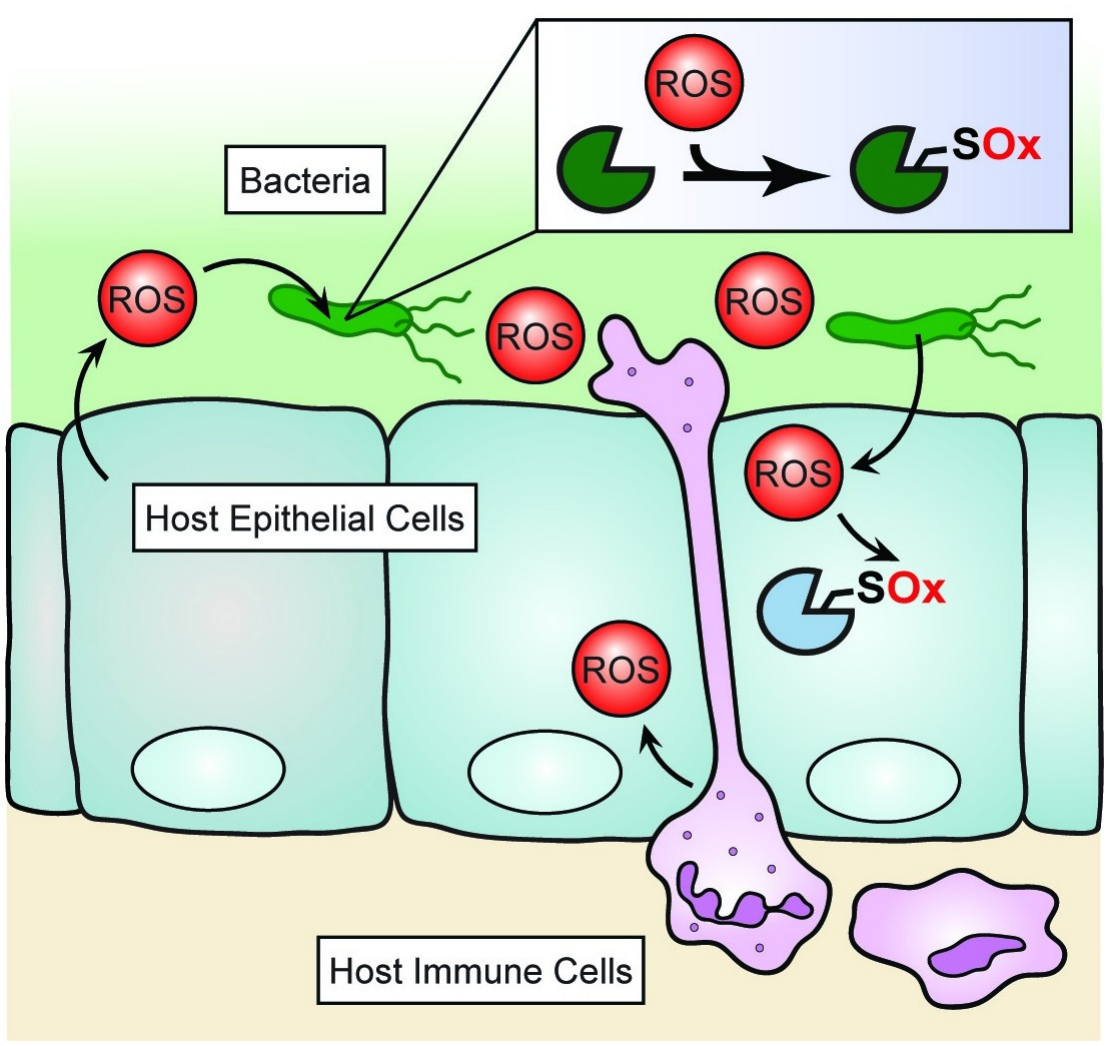
Redox signaling at the host–microbe interface. ROS produced by bacteria, host epithelial cells, and migratory immune cells can alter cell signaling via the site-specific Ox of proteins containing redox-sensitive cysteines. Ox, oxidation; ROS, reactive oxygen species.

Redox signaling occurs through the posttranslational oxidation of proteins containing redox-sensitive amino acids (Fig 1). OxiPTMs can significantly alter protein structure, function, and/or localization. Cysteine residues are arguably the most common targets of OxiPTMs [1]. At physiological pH, cysteine thiols (R-SH) with particularly low p*K*_a_s exist as nucleophilic thiolate anions (R-S^−^). Thiolates can react with ROS to yield a variety of OxiPTMs, including sulfenic (R-SOH), sulfinic (R-SO_2_H), and sulfonic (R-SO_3_H) acids, as well as disulfides (RS-SR). Other amino acids, like histidine, methionine, and tyrosine, are also susceptible to oxidation under physiological conditions [1,9]. Given the variety of amino acids that can be targeted by ROS, and the chemical diversity of the resulting OxiPTMs, specialized methods are required to detect these modifications. Here, we focus on mass spectrometry (MS)-based proteomic techniques for mapping cysteine OxiPTMs that contribute to redox signaling.

## Why are chemical approaches useful for detecting redox signaling?

Historically, transcriptional profiling and targeted biochemical analyses have been used to identify oxidation-inducible genes and redox-sensitive proteins. For example, quantitative thiol-disulfide titrations and targeted mutational analyses revealed that the formation of a disulfide bond in OxyR, a widely conserved bacterial transcription factor, induces the transcription of antioxidant enzymes under conditions of oxidative stress [10]. While similar analyses have been valuable for characterizing specific mechanisms of redox-regulated signaling in bacterial and host cells [9,11], they are not easily scalable to the entire proteome. In addition, because OxiPTMs occur posttranslationally, and many are short-lived, they cannot be directly detected using conventional profiling approaches. Recently developed chemical proteomic techniques complement traditional methods for uncovering redox-sensitive proteins by enabling the systematic identification of cysteines that are oxidized by ROS.

### Chemical proteomic techniques for identifying oxidized cysteines

**Fig 2:**
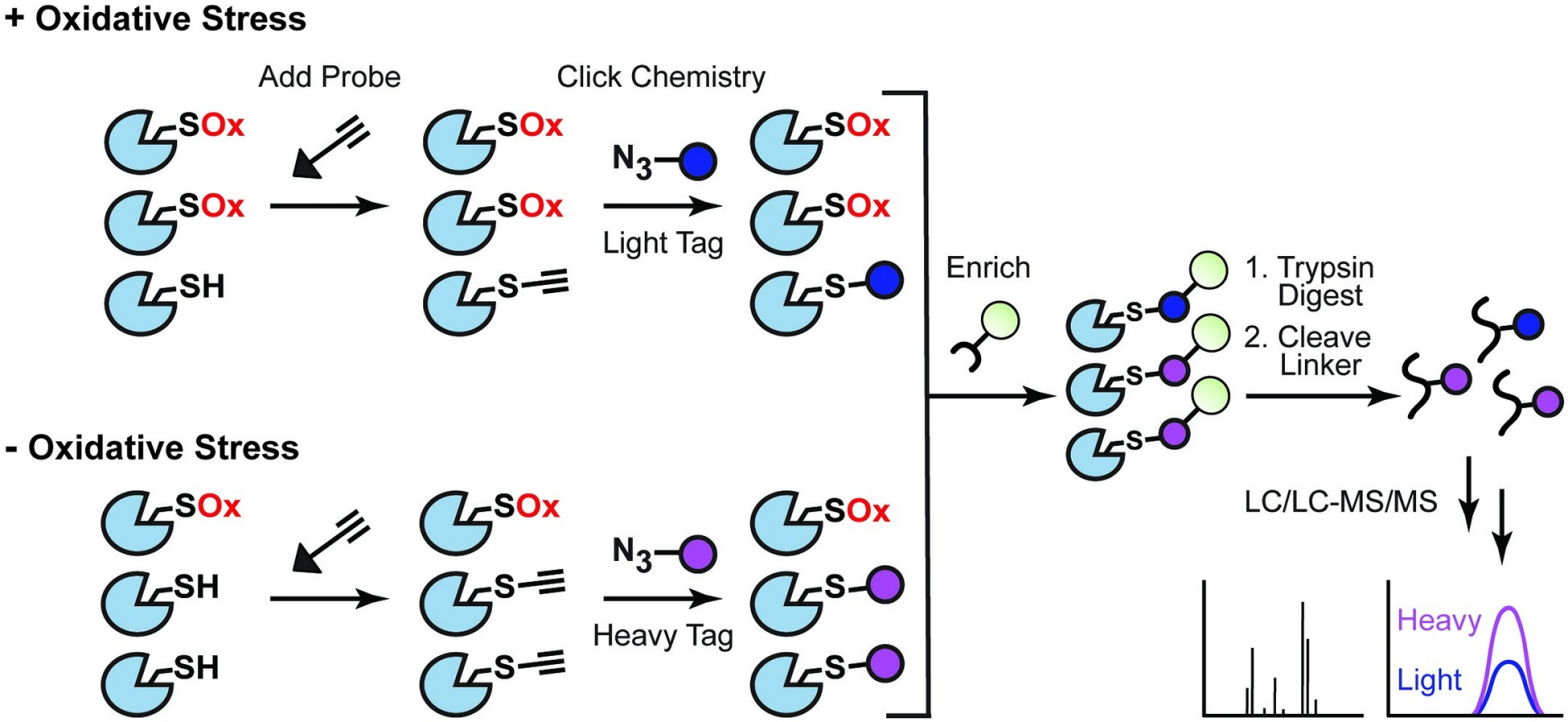
Globally profiling cysteine reactivity at the host–microbe interface using isoTOP–ABPP. Nucleophilic cysteines in each sample (e.g., cells treated with or without oxidative stress) are labeled with a thiol-reactive probe, which is then conjugated to an enrichment tag containing an isotopically labeled linker via click chemistry. Following enrichment and trypsin digestion of probe-labeled proteins, the relative abundance of probe-labeled peptides in each sample is quantified by MS. isoTOP–ABPP, isotopic tandem orthogonal proteolysis–activity-based protein profiling; LC, liquid chromatography; MS, mass spectrometry; Ox, oxidation.

Chemical proteomic methods rely on small-molecule, activity-based probes to facilitate the detection of reactive amino acids within a whole proteome. Probes that selectively label nucleophilic cysteine thiols can be used to globally profile changes in cysteine reactivity due to OxiPTMs, which inhibit probe labeling. By using liquid chromatography (LC)–MS to quantify the relative abundance of probe-labeled peptides under different sample conditions (e.g., cells grown in the presence or absence of oxidative stress), chemical proteomic techniques like isotopic tandem orthogonal proteolysis–activity-based protein profiling (isoTOP–ABPP) [12] and OxICAT [13] can resolve precise sites of cysteine oxidation. For example, in isoTOP–ABPP, reactive cysteines are labeled with a thiol-reactive iodoacetamide-alkyne probe, which is subsequently coupled to an isotopically labeled biotin-azide tag with a cleavable linker via copper-catalyzed azide–alkyne cycloaddition (CuAAC; AKA click chemistry) (Fig 2). Probe-labeled proteins are enriched using streptavidin beads and digested with trypsin. The remaining probe-labeled peptides are eluted from the beads via linker cleavage and quantified by LC/LC–MS/MS. The use of a unique isotopic tag for each sample enables the relative quantification of peptide abundance. Similarly, OxICAT employs isotope-coded iodoacetamide probes with cleavable linkers to detect cysteine OxiPTMs, but incorporates a reduction step into the sample processing workflow to distinguish reduced versus oxidized cysteines within a single sample [13]. First, the proteome is denatured and treated with an isotopically “light” probe to irreversibly label all reduced protein thiols; the sample is then incubated with a reducing agent to permit labeling of reversibly oxidized cysteines (e.g., disulfides) with an isotopically “heavy” probe. Following digestion and purification of the probe-labeled peptides, LC–MS/MS analysis is used to calculate the relative oxidation of individual cysteines within the proteome.

The indirect detection of oxidized cysteines using isoTOP–ABPP or OxICAT offers an unbiased approach to identifying OxiPTMs. Because these methods quantify proteome-wide changes in cysteine reactivity, they can detect a wide range of thiol-based modifications that inhibit probe labeling, irrespective of the type of modification. Methods for the direct detection of proteins containing specific classes of oxidized cysteines have also been developed [14]. These approaches rely on chemotype-specific probes that selectively label distinct classes of OxiPTMs (e.g., alkyne-functionalized dimedone probes for the detection of sulfenic acids and 2-nitroso benzoic acid derivatives for the detection of sulfinic acids [14,15]), thereby enabling the direct enrichment and MS-based identification of oxidized proteins. The expanding toolkit for profiling cysteine OxiPTMs, which also includes cell-permeable, photoactivatable probes for studies of transient oxidative modifications in living cells [16], provides new opportunities to investigate dynamic redox signaling in physiological systems.

### Mapping oxidized cysteines in bacterial and host cells

To date, MS-based approaches for identifying oxidized cysteines have primarily been used to study cells exposed to chemical oxidants in vitro. For example, both isoTOP–ABPP and OxICAT have uncovered redox-sensitive cysteines in hundreds of bacterial proteins under conditions of oxidative stress [13,17]. Several of the identified proteins, such as OxyR, contain redox-sensitive thiols with known roles in antioxidant defense, validating the ability of these approaches to detect functionally significant OxiPTMs within the proteome. Notably, many other proteins that were found to contain oxidized cysteines mediate critical processes beyond the antioxidant response, such as bacterial metabolism and DNA replication, suggesting that ROS may play a much broader role in regulating bacterial physiology than previously appreciated. Chemical proteomic tools have more recently been applied to identify cysteines oxidized by physiological sources of ROS. For example, OxICAT has been used to detect oxidized thiols in the proteome of phagocytosed *Escherichia coli* [18]. In addition, efforts to profile oxidized cysteines in the mucosa of monocolonized mice promise to expand our understanding of symbiotic interactions mediated by bacterial- and host-derived ROS in vivo [19].

## Future directions

ROS produced at the host–microbe interface are well positioned to modulate signaling pathways that underlie symbiosis or pathogenesis. Prior studies of bacteria exposed to oxidative stress in vitro have revealed hundreds of proteins that are specifically targeted by ROS [13,17]. Dissecting the contributions of protein oxidation to signal transduction in physiologically relevant systems, such as animal models of infection, will be necessary to understand the role of OxiPTMs in health and disease.

Many microbe-associated pathologies, such as gastrointestinal cancers, colitis, and peptic ulcers, have been linked to oxidative stress [3]. Oxidative stress can induce DNA damage and genomic instability, but far less is known about the role of protein oxidation in infection. Thus, many questions regarding how OxiPTMs influence disease progression remain unanswered: Does dysbiosis give rise to OxiPTMs that corrupt host cell signaling? Could novel therapeutics targeting redox-sensitive proteins ameliorate disease? Conversely, can mutualistic bacteria trigger redox-signaling pathways that confer health benefits to the host?

Chemical profiling technologies coupled with chemotype-specific probes should help establish the contribution of specific OxiPTMs to host–microbe interactions. Increasingly sophisticated infection models, such as germ-free animals and 3D organoids, will also facilitate the functional analysis of redox signaling in complex systems. A recent quantitative characterization of the mouse cysteine redox proteome demonstrates the feasibility of mapping tissue-specific oxidation events in vivo [20]. Harnessing these technologies to detect oxidative crosstalk at the host–microbe interface could uncover a vast network of ROS-mediated signaling pathways that shape bacterial and host physiology.
